# 

**Published:** 1892-02

**Authors:** 


					﻿Vol. XII.
FEBRUARY, 1892.
NO. 2;
THE
HOMEOPATHIC pHY^lClAfl,
A Monthly Journal of Homoeopathic Materia Medica
and Clinical Medicine.
*• He is a freeman whom truth makes free, and all are slaves beside.”
“Seek the Truth: come whence it may, cost what it will."
EDITED AND PUBLISHED BY
WALTER M. JAMES, NT. D.
PHILADELPHIA :
No. 1125 SPRUCE STREET.
LONEON :
ALFRED HEATH & CO., Sole Agents for Great Britain,
114 Ebury Street, Eaton Square.
JtSF Yearly Subscription, $2.50. invariably in advance. Single numbers, 25 cts.
Entered at the Philadelphia Peat-Office aa SeeonJ Claaa Mall Matter.
				

